# 2-Chloro-*N*-(2,3-dichloro­phen­yl)acetamide

**DOI:** 10.1107/S1600536808000408

**Published:** 2008-01-11

**Authors:** B. Thimme Gowda, Sabine Foro, Hartmut Fuess

**Affiliations:** a Department of Chemistry, Mangalore University, Mangalagangotri 574 199, Mangalore, India; b Institute of Materials Science, Darmstadt University of Technology, Petersenstrasse 23, D-64287, Darmstadt, Germany

## Abstract

The conformation of the N—H bond in the title compound (23DCPCA), C_8_H_6_Cl_3_NO, is *syn* to both the 2- and 3-chloro substituents in the aromatic ring, similar to the 2-chloro substituent in 2-chloro-*N*-(2-chloro­phen­yl)acetamide (2CPCA), the 2- and 3-chloro substituents in *N*-(2,3-dichloro­phen­yl)acetamide (23DCPA) and in 2,2-dichloro-*N*-(2,3-dichloro­phen­yl)acetamide (23DCPDCA). The bond parameters in 23DCPCA are similar to those in 2-chloro-*N*-(phen­yl)acetamide, 2CPCA, 23DCPA, 23DCPDCA and other acetanilides. The mol­ecules in 23DCPCA are linked into chains through N—H⋯O hydrogen bonding.

## Related literature

For related literature, see: Gowda *et al.* (2007*a* ▶,*b* ▶,*c* ▶); Shilpa & Gowda (2007 ▶).
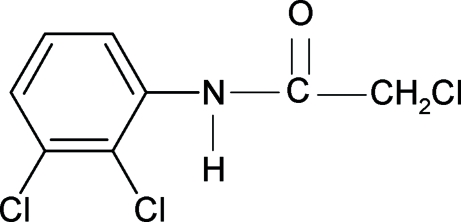


## Experimental

### 

#### Crystal data

C_8_H_6_Cl_3_NO
*M* *_r_* = 238.49
Monoclinic, 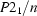

*a* = 11.704 (3) Å
*b* = 4.712 (1) Å
*c* = 17.503 (4) Å
β = 99.76 (2)°
*V* = 951.3 (4) Å^3^
*Z* = 4
Cu *K*α radiation
μ = 8.38 mm^−1^
*T* = 299 (2) K
0.50 × 0.35 × 0.28 mm

#### Data collection

Enraf–Nonius CAD-4 diffractometer
Absorption correction: ψ scan (North *et al.*, 1968 ▶) *T*_min_ = 0.014, *T*_max_ = 0.096
1832 measured reflections
1692 independent reflections
1625 reflections with *I* > 2σ(*I*)
*R*_int_ = 0.036
3 standard reflections frequency: 120 min intensity decay: 2.0%

#### Refinement

*R*[*F* ^2^ > 2σ(*F* ^2^)] = 0.080
*wR*(*F* ^2^) = 0.231
*S* = 1.07
1692 reflections
122 parameters
H atoms treated by a mixture of independent and constrained refinement
Δρ_max_ = 0.87 e Å^−3^
Δρ_min_ = −1.04 e Å^−3^

### 

Data collection: *CAD-4-PC* (Enraf–Nonius, 1996 ▶); cell refinement: *CAD-4-PC*; data reduction: *REDU4* (Stoe & Cie, 1987 ▶); program(s) used to solve structure: *SHELXS97* (Sheldrick, 2008 ▶); program(s) used to refine structure: *SHELXL97* (Sheldrick, 2008 ▶); molecular graphics: *PLATON* (Spek, 2003 ▶); software used to prepare material for publication: *SHELXL97*.

## Supplementary Material

Crystal structure: contains datablocks I, global. DOI: 10.1107/S1600536808000408/om2201sup1.cif

Structure factors: contains datablocks I. DOI: 10.1107/S1600536808000408/om2201Isup2.hkl

Additional supplementary materials:  crystallographic information; 3D view; checkCIF report

## Figures and Tables

**Table 1:** Hydrogen-bond geometry (Å, °)

*D*—H⋯*A*	*D*—H	H⋯*A*	*D*⋯*A*	*D*—H⋯*A*
N1—H1*N*⋯O1^i^	0.85 (5)	2.05 (5)	2.862 (4)	161 (4)

Symmetry code: (i) 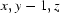.

**Fig. 1.**
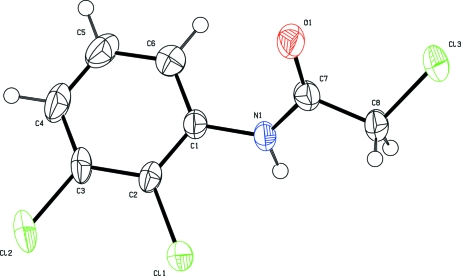
Molecular structure of the title compound, showing the atom labeling scheme. The displacement ellipsoids are drawn at the 50% probability level.

**Fig. 2.**
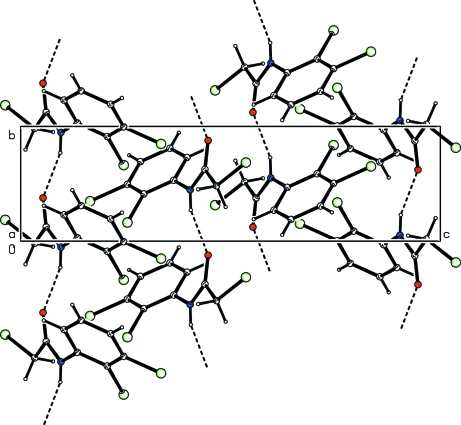
Molecular packing of the title compound with hydrogen bonding shown as dashed lines.

## supplementary crystallographic information

### Comment

In the present work, the structure of 2-chloro-*N*-(2,3-dichlorophenyl)- acetamide (23DCPCA) has been determined to study the effect of substituents on the structures of *N*-aromatic amides (Gowda *et al.*, 2007*a*, *b*, *c*). The conformation of the N—H bond in 23DCPCA is *syn* to both the 2-chloro and 3-chloro substituent (Fig. 1), similar to that of 2-chloro substituent in 2-chloro-*N*-(2-chlorophenyl)acetamide (2CPCA)(Gowda *et al.*, 2007*b*), 2- and 3-chloro substituents in *N*-(2,3-dichlorophenyl)-acetamide (23DCPA) (Gowda *et al.*, 2007*a*) and in 2,2-dichloro-*N*-(2,3-dichlorophenyl)acetamide (23DCPDCA)(Gowda *et al.*, 2007*c*). The bond parameters in 23DCPCA are similar to those in 2-chloro-*N*-(phenyl)acetamide, 2CPCA, 23DCPA, 23DCPDCA and other acetanilides (Gowda *et al.*, 2007*a*, *b*, *c*). The molecules in the structure of 23DCPCA are stabilized through N—H···O hydrogen bonding (Table 1 and Fig.2).

### Experimental

The title compound was prepared according to the literature method (Shilpa & Gowda, 2007). The purity of the compound was checked by determining its melting point. It was characterized by recording its infrared and NMR spectra (Shilpa & Gowda, 2007). Single crystals of the title compound were obtained from an ethanolic solution and used for X-ray diffraction studies at room temperature.

### Refinement

The CH atoms were positioned with idealized geometry using a riding model with C—H = 0.93–0.97 Å. The NH atom was located in difference map with N—H = 0.85 (5) Å. *U*_iso_(H) values were set equal to 1.2 *U*_eq_ of the parent atom.

The residual electron-density features are located in the region of Cl1. The highest peak and deepest hole are 1.04 and 0.80%A from Cl1, respectively.

### Crystal data

**Table:**

C_8_H_6_Cl_3_NO	*F*_000_ = 480
*M**_r_* = 238.49	*D*_x_ = 1.665 Mg m^−^^3^
Monoclinic, *P*2_1_/*n*	Cu *K*α radiation λ = 1.54180 Å
Hall symbol: -P 2yn	Cell parameters from 25 reflections
*a* = 11.704 (3) Å	θ = 4.3–23.9º
*b* = 4.712 (1) Å	µ = 8.38 mm^−^^1^
*c* = 17.503 (4) Å	*T* = 299 (2) K
β = 99.76 (2)º	Prism, colourless
*V* = 951.3 (4) Å^3^	0.50 × 0.35 × 0.28 mm
*Z* = 4	

### Data collection

**Table:**

Enraf–Nonius CAD-4 diffractometer	*R*_int_ = 0.036
Radiation source: fine-focus sealed tube	θ_max_ = 66.9º
Monochromator: graphite	θ_min_ = 4.2º
*T* = 299(2) K	*h* = −13→1
ω/2θ scans	*k* = −5→0
Absorption correction: ψ scan(North *et al.*, 1968)	*l* = −20→20
*T*_min_ = 0.014, *T*_max_ = 0.096	3 standard reflections
1832 measured reflections	every 120 min
1692 independent reflections	intensity decay: 2.0%
1625 reflections with *I* > 2σ(*I*)	

### Refinement

**Table:**

Refinement on *F*^2^	Hydrogen site location: inferred from neighbouring sites
Least-squares matrix: full	H atoms treated by a mixture of independent and constrained refinement
*R*[*F*^2^ > 2σ(*F*^2^)] = 0.080	*w* = 1/[σ^2^(*F*_o_^2^) + (0.181*P*)^2^ + 0.9328*P*] where *P* = (*F*_o_^2^ + 2*F*_c_^2^)/3
*wR*(*F*^2^) = 0.231	(Δ/σ)_max_ = 0.025
*S* = 1.07	Δρ_max_ = 0.87 e Å^−^^3^
1692 reflections	Δρ_min_ = −1.04 e Å^−^^3^
122 parameters	Extinction correction: SHELXL, Fc^*^=kFc[1+0.001xFc^2^λ^3^/sin(2θ)]^-1/4^
Primary atom site location: structure-invariant direct methods	Extinction coefficient: 0.018 (3)
Secondary atom site location: difference Fourier map	

### Special details

**Table:**

Geometry. All e.s.d.'s (except the e.s.d. in the dihedral angle between two l.s. planes) are estimated using the full covariance matrix. The cell e.s.d.'s are taken into account individually in the estimation of e.s.d.'s in distances, angles and torsion angles; correlations between e.s.d.'s in cell parameters are only used when they are defined by crystal symmetry. An approximate (isotropic) treatment of cell e.s.d.'s is used for estimating e.s.d.'s involving l.s. planes.
Refinement. Refinement of *F*^2^ against ALL reflections. The weighted *R*-factor *wR* and goodness of fit *S* are based on *F*^2^, conventional *R*-factors *R* are based on *F*, with *F* set to zero for negative *F*^2^. The threshold expression of *F*^2^ > σ(*F*^2^) is used only for calculating *R*-factors(gt) *etc*. and is not relevant to the choice of reflections for refinement. *R*-factors based on *F*^2^ are statistically about twice as large as those based on *F*, and *R*- factors based on ALL data will be even larger.

### Fractional atomic coordinates and isotropic or equivalent isotropic displacement parameters (Å^2^)

**Table:**

	*x*	*y*	*z*	*U*_iso_*/*U*_eq_	
C1	0.4488 (3)	0.5307 (7)	0.36495 (19)	0.0315 (8)	
C2	0.4556 (3)	0.4122 (7)	0.2935 (2)	0.0316 (8)	
C3	0.5442 (3)	0.4955 (9)	0.2544 (2)	0.0395 (9)	
C4	0.6240 (4)	0.6936 (10)	0.2854 (3)	0.0502 (11)	
H4	0.6825	0.7486	0.2586	0.060*	
C5	0.6172 (4)	0.8099 (9)	0.3558 (3)	0.0502 (11)	
H5	0.6714	0.9453	0.3768	0.060*	
C6	0.5313 (3)	0.7298 (9)	0.3964 (2)	0.0416 (9)	
H6	0.5284	0.8089	0.4447	0.050*	
C7	0.2947 (4)	0.6252 (8)	0.4390 (2)	0.0376 (9)	
C8	0.1935 (4)	0.4823 (8)	0.4667 (2)	0.0430 (10)	
H8A	0.2178	0.2983	0.4884	0.052*	
H8B	0.1320	0.4519	0.4228	0.052*	
N1	0.3582 (3)	0.4477 (7)	0.40382 (17)	0.0347 (8)	
H1N	0.346 (4)	0.271 (11)	0.405 (3)	0.042*	
O1	0.3132 (3)	0.8774 (6)	0.4466 (2)	0.0556 (9)	
Cl1	0.35541 (7)	0.16288 (19)	0.25447 (5)	0.0389 (5)	
Cl2	0.55325 (12)	0.3482 (3)	0.16544 (7)	0.0653 (6)	
Cl3	0.14026 (11)	0.6855 (3)	0.53663 (7)	0.0602 (5)	

### Atomic displacement parameters (Å^2^)

**Table:**

	*U*^11^	*U*^22^	*U*^33^	*U*^12^	*U*^13^	*U*^23^
C1	0.0320 (17)	0.0313 (17)	0.0342 (16)	−0.0005 (13)	0.0143 (13)	0.0009 (13)
C2	0.0245 (16)	0.0345 (17)	0.0383 (18)	−0.0019 (13)	0.0126 (13)	0.0041 (14)
C3	0.0316 (17)	0.050 (2)	0.0428 (19)	0.0012 (16)	0.0234 (14)	0.0043 (16)
C4	0.034 (2)	0.056 (2)	0.067 (3)	−0.0074 (17)	0.0248 (19)	0.006 (2)
C5	0.033 (2)	0.049 (2)	0.070 (3)	−0.0125 (17)	0.0109 (19)	−0.0012 (19)
C6	0.040 (2)	0.045 (2)	0.042 (2)	−0.0058 (16)	0.0097 (16)	−0.0031 (16)
C7	0.050 (2)	0.0314 (18)	0.0354 (18)	−0.0010 (15)	0.0188 (16)	0.0010 (14)
C8	0.048 (2)	0.040 (2)	0.049 (2)	−0.0055 (16)	0.0304 (17)	−0.0054 (16)
N1	0.0431 (16)	0.0293 (15)	0.0376 (16)	−0.0022 (12)	0.0236 (13)	−0.0005 (12)
O1	0.074 (2)	0.0305 (15)	0.076 (2)	−0.0035 (13)	0.0499 (18)	−0.0054 (13)
Cl1	0.0360 (7)	0.0433 (7)	0.0411 (7)	−0.0057 (3)	0.0170 (4)	−0.0078 (3)
Cl2	0.0670 (9)	0.0878 (10)	0.0529 (8)	−0.0123 (6)	0.0438 (6)	−0.0074 (5)
Cl3	0.0646 (9)	0.0640 (9)	0.0639 (8)	−0.0105 (5)	0.0454 (6)	−0.0179 (5)

### Geometric parameters (Å, °)

**Table:**

C1—C2	1.384 (5)	C5—H5	0.9300
C1—C6	1.391 (5)	C6—H6	0.9300
C1—N1	1.409 (4)	C7—O1	1.211 (5)
C2—C3	1.392 (5)	C7—N1	1.337 (5)
C2—Cl1	1.718 (3)	C7—C8	1.512 (5)
C3—C4	1.366 (6)	C8—Cl3	1.750 (4)
C3—Cl2	1.725 (4)	C8—H8A	0.9700
C4—C5	1.363 (7)	C8—H8B	0.9700
C4—H4	0.9300	N1—H1N	0.85 (5)
C5—C6	1.379 (6)		
			
C2—C1—C6	119.2 (3)	C5—C6—C1	119.9 (4)
C2—C1—N1	119.2 (3)	C5—C6—H6	120.0
C6—C1—N1	121.7 (3)	C1—C6—H6	120.0
C1—C2—C3	119.5 (3)	O1—C7—N1	124.2 (4)
C1—C2—Cl1	119.7 (3)	O1—C7—C8	122.5 (4)
C3—C2—Cl1	120.8 (3)	N1—C7—C8	113.3 (3)
C4—C3—C2	120.9 (4)	C7—C8—Cl3	111.8 (3)
C4—C3—Cl2	119.4 (3)	C7—C8—H8A	109.3
C2—C3—Cl2	119.7 (3)	Cl3—C8—H8A	109.3
C5—C4—C3	119.5 (4)	C7—C8—H8B	109.3
C5—C4—H4	120.2	Cl3—C8—H8B	109.3
C3—C4—H4	120.2	H8A—C8—H8B	107.9
C4—C5—C6	121.0 (4)	C7—N1—C1	124.9 (3)
C4—C5—H5	119.5	C7—N1—H1N	119 (3)
C6—C5—H5	119.5	C1—N1—H1N	116 (3)
			
C6—C1—C2—C3	−0.3 (5)	C3—C4—C5—C6	0.2 (7)
N1—C1—C2—C3	179.3 (3)	C4—C5—C6—C1	−1.0 (7)
C6—C1—C2—Cl1	179.3 (3)	C2—C1—C6—C5	1.0 (6)
N1—C1—C2—Cl1	−1.1 (5)	N1—C1—C6—C5	−178.6 (4)
C1—C2—C3—C4	−0.4 (6)	O1—C7—C8—Cl3	−20.3 (5)
Cl1—C2—C3—C4	−180.0 (3)	N1—C7—C8—Cl3	161.7 (3)
C1—C2—C3—Cl2	179.9 (3)	O1—C7—N1—C1	−5.7 (6)
Cl1—C2—C3—Cl2	0.3 (5)	C8—C7—N1—C1	172.3 (3)
C2—C3—C4—C5	0.4 (6)	C2—C1—N1—C7	−135.6 (4)
Cl2—C3—C4—C5	−179.9 (3)	C6—C1—N1—C7	44.0 (5)

### Hydrogen-bond geometry (Å, °)

**Table:**

*D*—H···*A*	*D*—H	H···*A*	*D*···*A*	*D*—H···*A*
N1—H1N···O1^i^	0.85 (5)	2.05 (5)	2.862 (4)	161 (4)

Symmetry codes: (i) *x*, *y*−1, *z*.

## References

[bb1] Enraf–Nonius (1996). *CAD-4-PC.* Version 1.2. Enraf–Nonius, Delft, The Netherlands.

[bb2] Gowda, B. T., Foro, S. & Fuess, H. (2007*a*). *Acta Cryst.* E**63**, o2631–o2632.

[bb3] Gowda, B. T., Foro, S. & Fuess, H. (2007*b*). *Acta Cryst.* E**63**, o4611.

[bb4] Gowda, B. T., Foro, S. & Fuess, H. (2007*c*). *Acta Cryst.* E**63**, o4708.

[bb5] North, A. C. T., Phillips, D. C. & Mathews, F. S. (1968). *Acta Cryst.* A**24**, 351–359.

[bb6] Sheldrick, G. M. (2008). *Acta Cryst.* A**64**, 112–122. 10.1107/S0108767307043930 18156677

[bb7] Shilpa & Gowda, B. T. (2007). *Z. Naturforsch. Teil A*, **62**, 84–90.

[bb8] Spek, A. L. (2003). *J. Appl. Cryst.* **36**, 7–13.

[bb9] Stoe & Cie (1987). *REDU4* Version 6.2c. Stoe & Cie GmbH, Darmstadt, Germany.

